# Emergence of Resistance to Fluoroquinolones and Third-Generation Cephalosporins in *Salmonella* Typhi in Lahore, Pakistan

**DOI:** 10.3390/microorganisms8091336

**Published:** 2020-09-01

**Authors:** Farhan Rasheed, Muhammad Saeed, Nabil-Fareed Alikhan, David Baker, Mohsin Khurshid, Emma V. Ainsworth, A. Keith Turner, Ambereen Anwar Imran, Muhammad Hidayat Rasool, Muhammad Saqalein, Muhammad Atif Nisar, Muhammad Fayyaz ur Rehman, John Wain, Muhammad Yasir, Gemma C. Langridge, Aamer Ikram

**Affiliations:** 1 Allama Iqbal Medical College & Jinnah Hospital (AIMC&JHL), Lahore 54000, Pakistan; ambereen@outlook.com; 2 Department of Microbiology, Government College University, Faisalabad 38000, Pakistan; mian.muhsaeed@gmail.com (M.S.); mohsinkhurshid@gcuf.edu.pk (M.K.); drmhrasool@gcuf.edu.pk (M.H.R.); drsaqalein@gcuf.edu.pk (M.S.); matifnisar@gcuf.edu.pk (M.A.N.); 3 Quadram Institute Bioscience, Norwich Research Park, Norwich NR4 7UQ, UK; nabil-fareed.alikhan@quadram.ac.uk (N.-F.A.); david.baker@quadram.ac.uk (D.B.); emma.ainsworth@quadram.ac.uk (E.V.A.); keith.turner@quadram.ac.uk (A.K.T.); john.wain@quadram.ac.uk (J.W.); gemma.langridge@quadram.ac.uk (G.C.L.); 4 Department of Chemistry, University of Sargodha, Sargodha 40100, Pakistan; fayyaz9@gmail.com; 5 Norwich Medical School, University of East Anglia, Norwich Research Park, Norwich NR4 7TJ, UK; 6 National Institute of Health, Islamabad 45710, Pakistan; maahin1@yahoo.com

**Keywords:** *Salmonella* Typhi, typhoid fever, XDR, cephalosporin resistance, Lahore

## Abstract

Extensively drug-resistant (XDR) *Salmonella* Typhi has been reported in Sindh province of Pakistan since 2016. The potential for further spread is of serious concern as remaining treatment options are severely limited. We report the phenotypic and genotypic characterization of 27 XDR *S*. Typhi isolated from patients attending Jinnah Hospital, Lahore, Pakistan. Isolates were identified by biochemical profiling; antimicrobial susceptibility was determined by a modified Kirby–Bauer method. These findings were confirmed using Illumina whole genome nucleotide sequence data. All sequences were compared to the outbreak strain from Southern Pakistan and typed using the *S*. Typhi genotyping scheme. All isolates were confirmed by a sequence analysis to harbor an IncY plasmid and the CTX-M-15 ceftriaxone resistance determinant. All isolates were of the same genotypic background as the outbreak strain from Sindh province. We report the first emergence of XDR *S*. Typhi in Punjab province of Pakistan confirmed by whole genome sequencing.

## 1. Introduction

Typhoid fever, caused by *Salmonella enterica*, serotype Typhi (*Salmonella* Typhi) is a global health concern. The burden of typhoid has been estimated at 13.5 million cases resulting in 135,000 deaths annually, with a global incidence of 2.14 in 1000 [1,2]. In Pakistan, the precise burden is not known but active surveillance studies in Karachi report 4.7 cases per 1000 per year. Travel-associated typhoid fever is also of concern in high income countries with more than 150 cases of typhoid fever annually in England and Wales since 2008 (Public Health England, 2018); most of these cases have a history of travel to South Asia. The treatment of typhoid was changed by the emergence of multidrug resistance (MDR) in *Salmonella* Typhi, which rendered first line antibiotics (amoxycillin, co-trimoxazole, and chloramphenicol) and fluoroquinolones ineffective [3]. The treatment of choice became a third-generation cephalosporin, most commonly ceftriaxone [4]. In 2016, there was a report from Karachi (Sindh province) of the emergence of extensively drug resistant (XDR) *S*. Typhi [5]. The *S*. Typhi strains described as XDR are MDR strains with additional fluoroquinolone and ceftriaxone resistance, leaving azithromycin, piperacillin-tazobactam, or carbapenem as the treatment options [5]. While a report of fluoroquinolone or ceftriaxone resistance in the Enterobacteriaceae is not uncommon in Pakistan, the isolation of *S*. Typhi harboring resistance to these antibiotics is much more unusual.

Since the first outbreak in Sindh, more than 17,000 XDR *S*. Typhi cases have been reported from this province according to National Institute of Health, Pakistan [6]. Prevalence data for XDR *S.* Typhi is not yet officially available outside Sindh province but there are case reports across the country [7,8], from elsewhere in the region [9] and it is being further transmitted by travelers [10]. In the current SARS-CoV-2 pandemic, a large number of typhoid fever cases have arisen with similar clinical fever symptoms to COVID-19; more than 20,000 typhoid cases were reported in June 2020 in Pakistan [11].

Whole genome sequencing (WGS) has been used in the past to understand the evolution of antibiotic resistance mechanisms and to track bacterial and viral pathogen spread [12,13]. WGS of individual cases of XDR *S.* Typhi from Taiwan, Canada and Denmark have shown a close relationship (100% nucleotide match) to the nearest sequenced XDR isolate from Pakistan [14,15,16]. This highlights the international impact of this ongoing outbreak and that any SNPs found between isolates of *S.* Typhi during an outbreak suggest prolonged transmission. This is the first study to use WGS for XDR *S.* Typhi found in Pakistan outside of Sindh province, and was prompted by the blood culture isolation of XDR *S*. Typhi from patients under investigation at Jinnah Hospital, Lahore. This raised the possibility that the Sindh province strain was circulating in Lahore, leading us to sequence 27 XDR *S*. Typhi isolates obtained in under 4 months in 2019 and compare SNPs in their whole genome sequences with those from Sindh.

## 2. Materials and Methods

This study was carried out at the Department of Pathology, Jinnah Hospital, Lahore, Pakistan and Quadram Institute Bioscience, Norwich, United Kingdom.

### 2.1. Isolation and Identification of Isolates

Blood cultures were collected, following normal diagnostic protocols, from patients with fever admitted to the Jinnah Hospital Lahore, Pakistan. A Gram stain was performed directly from blood culture broth; the broth was inoculated on blood agar and MacConkey agar. Bacterial colonies were identified biochemically using API 10 or API 20E (BioMérieux, Marcy l’Etoile, France). Isolates were confirmed as *Salmonella* Typhi by serological reactions to O, H, and Vi antigens (Pro-Lab Diagnostics, Ontario, Canada). Antibiotic susceptibility testing was performed using a modified Kirby–Bauer method according to Clinical Laboratory Standard Institute (CLSI) 2019 guidelines. Twenty-seven XDR *S*. Typhi isolates were collected between January 2019 and April 2019 and archived at −80 °C. The isolates were recultured on blood agar and DNA was extracted for sequencing from single colonies. Not all of the isolates seen were XDR but as blood cultures were from patients who presented themselves to health care services the data is not representative of the proportion of different strains circulating.

### 2.2. DNA Extraction

DNA extraction of *S*. Typhi isolates was carried out using a modified protocol of the PuriSpin Fire Monkey kit (RevoluGen, Hadfield, UK). In brief, 1 mL overnight *S*. Typhi culture was harvested and cells were resuspended in 100 µL of lysozyme (3 mg/mL), 1.2 % Triton X-100, and incubated at 37 °C, 180 rpm for 10 min. Of lysis solution 300 µL and 20 µL of proteinase K (20 mg/mL, Qiagen, Manchester, UK) was added to the partly lysed cells and incubated at 56 °C for 20 min. After lysis, 10 µL of RNase A (20 µg/µL, Sigma) was added to the suspension and incubated for a further 10 min at 37 °C. Binding solution 350 µL and isopropanol (75%) 400 µL were added to the lysed cells and the lysed cells were transferred to the spin column. Bound DNA was washed as per manufacturer’s instructions and eluted in 2 × 100 µL of elution buffer. DNA was shipped to Quadram Institute Bioscience, Norwich, UK and concentrations were determined using the Qubit dsDNA broad range assay kit (Thermo Fisher, Loughborough, UK) on a Qubit 3.0 Fluorometer (Thermo Fisher).

### 2.3. Whole Genome Nucleotide Sequencing

Genomic DNA concentrations were adjusted to 0.5 ng/µL with nuclease-free water for sequencing. A tagmentation mix of 3 µL was mixed with 2 µL of genomic DNA (0.5 ng/µL) and incubated at 55 °C for 10 min in a PCR block. Kapa2G PCR master mix was added 11 µL to each well sample in a 96-well plate alongside 2 µL of each of P7 and P5 of Nextera XT Index Kit v2 index primers (Illumina). The PCR was carried out following standard Illumina guidelines. The DNA fragment libraries were quantified using the Quant-iT dsDNA high sensitivity assay kit (Thermo Fisher) and pooled. The final pool was double-SPRI size selected between 0.5 and 0.7 × bead volumes using KAPA Pure Beads (Roche) and the pool was run at a final concentration of 1.8 pM on an Illumina Nextseq 500 instrument using a Mid Output Flowcell (NSQ^®^ 500 Mid Output KT v2 (300 CYS), Illumina). Data was uploaded to Basespace (www.basespace.illumina.com) where the raw data was converted to 8 FASTQ files for each sample.

### 2.4. Genome Assembly and Annotation

Genome nucleotide sequences of all strains were determined using 150 bp paired-end sequencing on the Illumina Nextseq 500 platform. All raw sequence data have been deposited in the NCBI Sequence Read Archive (SRA) under BioProject accession PRJNA602421. Sequenced reads were screened using Centrifuge v1.0.4 to confirm the species of each sample as *Salmonella enterica* [17]. De novo assembly of individual genomes was carried out using Shovill v1.0.0 (https://github.com/tseemann/shovill, accessed on 1 November 2019), which internally corrected sequencing errors, performed genome assembly using SPAdes [18], and removed erroneous contigs. Assembly quality was assessed via QUAST v5.0.2 [19,20]. Draft genomes were annotated using Prokka v1.14 [21].

### 2.5. Genotyping and Antimicrobial Gene Prediction

In silico multilocus sequence typing was predicted using “MLST” v2.17.5 (https://github.com/tseemann/mlst, accessed on 1 November 2019) using the *Salmonella* MLST database hosted by EnteroBase [22]. To calculate the genotype using the Genotyphi scheme [23], draft genomes were aligned using Parsnp v1.2 [13] to the *S.* Typhi CT18 reference genome (accession no. AL513382.1) and resulting variant calls (vcf) were run through Genotyphi (commit version fd1d58b), as described on the GitHub repository. Antimicrobial resistance genes were detected using ARIBA v2.14 [24] with default parameters.

### 2.6. Phylogenetic Analysis

Ninety-two additional *S*. Typhi genomes were included to provide context to the 27 from this study [5]. The outgroup and reference genome (ERL12148, accession no. LT883153.1) was selected using the following criteria; the strain was closely related (i.e., *S*. Typhi haplotype H58) and the strain was not associated with the 2016 outbreak. Single nucleotide polymorphisms (SNPs) were identified against the reference using Snippy v4.2.1 (https://github.com/tseemann/snippy, accessed on 1 November 2019) and visually inspected in Artemis [25]. Gubbins v2.3.1 [26] was used to define non-recombinant SNPs, which were used with RaxML [27] to construct the final phylogeny. A reference IncY plasmid (Pak60006-2016) [5] was used with Blast Ring Image Generator (BRIG) [28] to identify nucleotide identity between the reference genome and strains genome sequenced as part of this study.

### 2.7. Role of the Funding Source

The funders had no role in study design, in the collection, analysis and interpretation of data, in the writing of the report or the decision to submit this paper for publication.

### 2.8. Ethics Statement

Ethical approval for this study was obtained from the ethical review board of Allama Iqbal Medical College/Jinnah Hospital, Lahore at the 47th meeting held on 13 March 2019. All patient data was anonymized to remove any identifying information.

## 3. Results

### 3.1. Phenotypic Findings

Although strict surveillance data is not available for typhoid fever in Lahore, a 12-fold increase in isolation rate was observed at Jinnah Hospital over the 12-month period July 2018 to June 2019 (number of cases = 370) relative to the previous 12 months (*n* < 30). Twenty-seven isolates of *S*. Typhi were isolated from the blood of consecutive patients between 14th January and 1st April 2019. Fourteen isolates were from males and thirteen from females. The age range varied from 15 months to 28 years, with 11 of the isolates from under-fives, 13 from 5–16 years old, and 3 from >16 years old (Table S1). This was a typical sample of patients presenting to Jinnah Hospital with suspected typhoid fever. All of the isolates were phenotypically and biochemically identified as *S*. Typhi and serologically confirmed as positive to O, Vi, and H antibodies. Antibiotic susceptibility testing showed that these isolates were susceptible only to azithromycin, carbapenems, and piperacillin-tazobactam (Table S2). All were resistant to the standard treatment for typhoid fever including ceftriaxone. For all patients, treatment was started with intravenous meropenem followed by azithromycin. There was no mortality reported due to XDR *S*. Typhi.

### 3.2. Genotypic Analysis

**Figure 1:**
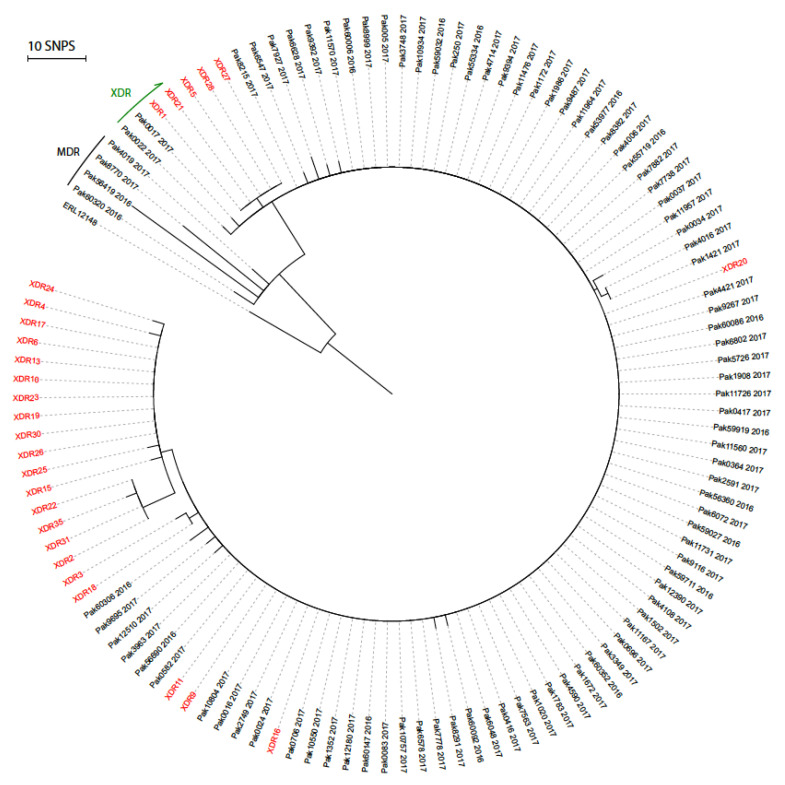
Phylogenetic tree of XDR *S*. Typhi. Radial phylogram of maximum-likelihood phylogeny (RaxML) rooted to *S*. Typhi ERL12148 (accession LT883153.1). PAK strain numbers in black are from [5] and XDR strain numbers (red) are from Lahore (this study). Black bar labeled MDR, indicates MDR strains and green arrow indicates that all strains from PAK0022 clockwise to XDR24 are XDR.

We compared our recent Lahore isolates to 100 *S*. Typhi genomes from Southern Pakistan and generated a maximum-likelihood phylogenetic tree from 68 filtered and neutral SNPs (Figure 1). All the XDR isolates differed by six SNPs from the MDR *S*. Typhi clade, the same level of variation as seen during the 2016 outbreak. Our Lahore isolates also intermingled with those from the 2016 outbreak, indicating few, if any, SNP differences between these strains. It is plausible therefore that all the XDR isolates originated from a single strain that acquired the XDR phenotype. Within the XDR clade, the Lahore isolates formed two clusters on the tree, one consisting of isolates XDR1, 21, 5, 28, and 27, and the other of XDR24, 4, 17, 6, 13, 10, 23, 19, 30, 26, 25, 15, 22, 35, 31, and 2, suggesting probable epidemiological linkage.

**Figure 2:**
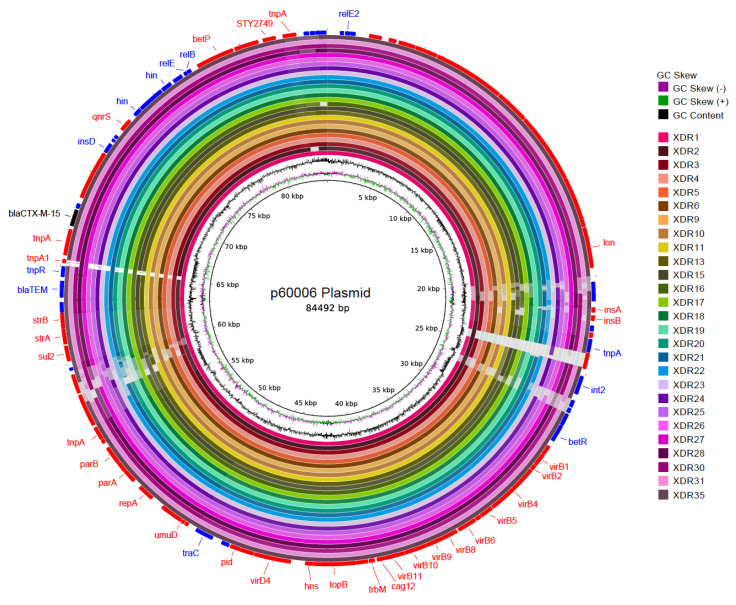
CTX-M-15 located on the IncY plasmid. The inner circle represents the reference plasmid sequence p60006. Concentric rings indicate BLAST identity between reference and strains sequenced as part of this study. BLASTn matches between 90% and 100% nucleotide identity are colored from lightest to darkest shade, respectively. Matches with less than 80% identity, or plasmid regions with no BLAST matches, appear as blank spaces in each ring. Inner most rings plot GC Skew and GC content. Outermost ring shows locations of coding sequences (red—sense; blue—antisense) and gene names are labeled where known. The CTX-M-15 gene is labeled in black.

Analysis of all 27 draft genome sequences from Lahore indicated the presence of both an IncY plasmid and the CTX-M-15 determinant associated with ceftriaxone resistance. The CTX-M-15 resistance determinant was located on an IncY plasmid in the Southern Pakistan outbreak strains; our BRIG analysis suggested that this was also the case for the Lahore isolates (Figure 2).

## 4. Discussion

Here we reported *S*. Typhi resistant to amoxycillin, co-trimoxazole, chloramphenicol, ciprofloxacin, and ceftriaxone from Lahore, Pakistan. Our phenotypic and genotypic analysis shows that the *S*. Typhi XDR isolates were indistinguishable from the outbreak strain described in Southern Pakistan, indicating that the cases in Lahore are part of the same outbreak. Whole genome nucleotide sequences revealed that all isolates were sequence type (ST)1 and identified as 4.3.1.1.P1 under the Genotyphi scheme, the same as the strains from the 2016 outbreak reported in Sindh province [5,23]. We have investigated a single tertiary health care facility in Lahore but alongside other reports, we believe it is spreading locally [29]. The isolates from Jinnah Hospital are closely related but several of them harbor SNP differences. Since *S*. Typhi is a clonal pathogen the presence of these SNPs suggests that either the XDR strain is not a recent introduction to the area or that it has been imported several times [30]. The emergence of XDR *S.* Typhi in Lahore is likely the result of regular travel between Sindh, Karachi, and Lahore encouraging national transmission, followed by the use of inactive antibiotics allowing local transmission.

The increased level of *S*. Typhi isolation from blood culture is of serious public health concern. Treatment of XDR *S*. Typhi is one of the growing challenges for infectious disease physicians in Lahore and more widely in Pakistan. Treatment is a challenge because a typical typhoid patient is from a poor socioeconomic background and the treatment of XDR is expensive. In Pakistan many adult patients present to their GP or pharmacist and are treated empirically whereas children are more often brought to the hospital [31]. The only treatment options that remain in this situation are azithromycin, meropenem, and piperacillin/tazobactam. The treatment strategy for XDR typhoid is intravenous meropenem for seven days, which costs 260 USD followed up by oral azithromycin; this is being used in Pakistan as well as in internationally exported cases [16] but many patients skip treatment and return later with complications (Asst. Prof. Fahad Aman Khan, personal communication). Carbapenems are a potent class of antibiotics and an effective treatment for typhoid fever but the presence of carbapenemase-producing Enterobacteriaceae is a critical threat [16,32]. This leaves many patients with azithromycin as their only viable option (Asst. Prof. Fahad Aman Khan, personal communication) [33].

Furthermore, of the remaining treatment options for XDR *S*. Typhi, only azithromycin is administered orally but the present danger is that mass administration of azithromycin for typhoid in addition to prophylactic use in COVID-19 management may accelerate the emergence and spread of azithromycin resistant XDR *S.* Typhi strains [34]. Resistance has already been reported in typhoidal *Salmonella*, conferred by single nucleotide changes in the *acrB* efflux system, from Bangladesh [35] and Pakistan [36], and the macrolide resistance genes *mphA*, *mphB,* and *mefB* have been reported as conferring resistance in non-typhoidal *Salmonella* [37]. It is likely only a matter of time before one of these mechanisms emerges in the XDR background, rendering carbapenems the only, more expensive, treatment option. It is also of note that while azithromycin remains a viable treatment in susceptible XDR isolates, it has been contraindicated for patients on arrhythmic, antipsychotics, and citalopram medications leading to an increased risk of cardiovascular-related mortality [38].

In conclusion, the risk of single point mutations conferring resistance to azithromycin and of the horizontal transmission of carbapenemase-mediated resistance makes the ongoing threat of XDR typhoid fever very real in Pakistan and internationally. One significant advancement towards treatment of typhoid in Pakistan is a program under development to provide the newly developed typhoid vaccine, Typbar-TCV, to more than 1 million children (6 month to 10 years of age) across infected areas [39]. This will help reduce the prevalence of typhoid but the efficacy of this vaccine against continuously emerging XDR typhoid will require evaluation for the next few years. Both epidemiological studies and functional genomics are urgently needed to inform local, national, and international control measures and future public health strategies.

## Supplementary Materials

The following are available online at https://www.mdpi.com/2076-2607/8/9/1336/s1, Table S1: Anonymised patient data. Table S2: Antimicrobial susceptibility testing results.
